# Transcriptionally active LTR retrotransposons in *Eucalyptus* genus are differentially expressed and insertionally polymorphic

**DOI:** 10.1186/s12870-015-0550-1

**Published:** 2015-08-14

**Authors:** Helena Sanches Marcon, Douglas Silva Domingues, Juliana Costa Silva, Rafael Junqueira Borges, Fábio Filippi Matioli, Marcos Roberto de Mattos Fontes, Celso Luis Marino

**Affiliations:** Departamento de Genética, Instituto de Biociências, Universidade Estadual Paulista – UNESP, Botucatu, Brazil; Programa de Pós-graduação em Ciências Biológicas (Genética), Universidade Estadual Paulista – UNESP, Botucatu, Brazil; Departamento de Botânica, Instituto de Biociências, Universidade Estadual Paulista – UNESP, Rio Claro, Brazil; Plant Biotechnology Laboratory, Instituto Agronômico do Paraná – IAPAR, Londrina, Brazil; Departamento de Física e Biofísica, Instituto de Biociências, Universidade Estadual Paulista – UNESP, Botucatu, Brazil and INCTTOX-CNPq, Brazil; Instituto de Biotecnologia da UNESP – IBTEC, Botucatu, Brazil

**Keywords:** LTR retrotransposons, Dynamics, Genomic distribution, Comparative analysis, *Eucalyptus* genomes

## Abstract

**Background:**

In *Eucalyptus* genus, studies on genome composition and transposable elements (TEs) are particularly scarce. Nearly half of the recently released *Eucalyptus grandis* genome is composed by retrotransposons and this data provides an important opportunity to understand TE dynamics in *Eucalyptus* genome and transcriptome.

**Results:**

We characterized nine families of transcriptionally active LTR retrotransposons from *Copia* and *Gypsy* superfamilies in *Eucalyptus grandis* genome and we depicted genomic distribution and copy number in two *Eucalyptus* species. We also evaluated genomic polymorphism and transcriptional profile in three organs of five *Eucalyptus* species. We observed contrasting genomic and transcriptional behavior in the same family among different species. RLC_*egMax*_1 was the most prevalent family and RLC_*egAngela*_1 was the family with the lowest copy number. Most families of both superfamilies have their insertions occurring <3 million years, except one *Copia* family, RLC_*egBianca*_1. Protein theoretical models suggest different properties between *Copia* and *Gypsy* domains. IRAP and REMAP markers suggested genomic polymorphisms among *Eucalyptus* species. Using EST analysis and qRT-PCRs, we observed transcriptional activity in several tissues and in all evaluated species. In some families, osmotic stress increases transcript values.

**Conclusion:**

Our strategy was successful in isolating transcriptionally active retrotransposons in *Eucalyptus*, and each family has a particular genomic and transcriptional pattern. Overall, our results show that retrotransposon activity have differentially affected genome and transcriptome among *Eucalyptus* species.

**Electronic supplementary material:**

The online version of this article (doi:10.1186/s12870-015-0550-1) contains supplementary material, which is available to authorized users.

## Background

Retrotransposons correspond to class I transposable elements, inserting into a host genome through an RNA intermediate [1]. Based on structural features and phylogenetic relationships, five orders of retrotransposons were defined [1]. LTR retrotransposons (LTR-RTEs), an order related to retroviruses, usually encode two open reading frames (ORFs): one called *gag*, which encodes a structural protein for virus-like particles, and another called *pol*, which encodes enzymatic domains involved in the transposition cycle, such as an aspartic protease, a reverse transcriptase, an RNaseH and an integrase [1]. The two major superfamilies of plant LTR retrotransposons are *Copia* and *Gypsy*, in which *pol* genes differ in their domain order [1].

In most angiosperm genomes, the LTR-RTEs are the most significant contributor to genome size, contributing over 70 % of the nuclear DNA in grasses like maize [2]. Most LTR retrotransposons families exist in low copy numbers, but the amplification of few individual families contribute with large differences in genome size among closely related species [3, 4].

Despite their stringent regulation, LTR-RTEs are transcriptionally active in plants [5–7]. Although epigenetic regulation is an important feature of most plant transposable elements [reviewed in [8], cis-regulation has a crucial role regulating LTR-RTE transcription, since LTRs represent promoter sequences. The modulation of LTR-RTEs transcriptional levels has been observed in different tissues, organs and development stages i.e. [5, 9].

The eucalypts are the most widely planted hardwoods in the world due to their ability to adapt, grow and provide quality wood for multiple applications [10]. Species of subgenus *Symphyomyrtus* account for > 95 % of the world's planted eucalypts [11]. These include three members of section Latoangulatae: *E. grandis*, *E. urophylla* and *E. saligna*, broadly planted in tropical areas due to their fast growth and disease resistance; and *E. tereticornis* (section Exsertaria), known for their drought tolerance and rapid growth [10]. Other species are better known for their potential for introgressing new traits in breeding, i.e., *E. brassiana* [12].

The *Eucalyptus grandis* genome assembly into 11 pseudochromosomes (605 megabases (Mb)), is composed by 44.5 % of retrotransposons, and LTR-RTEs are the most representative, constituting 21.9 % of the *E. grandis* genome [13]. Up to now, TE dynamics were scarcely studied in the *Eucalyptus* genus and most analysis were based on private EST data [i.e. [14]. In the present study, we identify and comprehensively characterize a selected group of *Eucalyptus* LTR-RTEs, emphasizing the characterization of elements with putative transcriptional activity. We analyzed the phylogenetic pattern of nine *E. grandis* LTR-RTEs families and we extended this analysis, understanding DNA interaction properties of selected encoding domains. Comparative classifications of LTR-RTEs from closely related species were performed on monocots in which transposable elements (TEs) had previously been well characterized [15, 16], however this is the first time this approach was employed in forest trees. Our study is the first to exhaustively sample and classify transcriptionally active TEs in *Eucalyptus* species, identifying their structure, genomic distribution, insertion time estimation, genomic polymorphism and transcriptional activity in five *Eucalyptus* species and one intrageneric hybrid.

## Methods

### *Eucalyptus grandis* transcriptionally active LTR-RTEs: selection and annotation

LTR-RTEs of *Copia* and *Gypsy* superfamilies described in Wicker and Keller [17] and Lloréns et al. [18] were used as queries in a BLASTX against *Eucalyptus* spp ESTs from the dbEST database at the National Center for Biotechnology Information (NCBI), website on 10/10/2011. We selected ESTs that aligned over 200 bp or more (e-value <1e − 50) for further analyses, similar to Rossi et al. [19]. In order to confirm whether the EST codes for a LTR-RTE, we analyzed sequences using CENSOR implemented in RepBase [20] and the ones where the LTR-RTE sequence matched more than 80 % of an EST were selected for other analyses.

These selected ESTs were used as queries in a BLASTN search to identify full-length LTR-RTEs in *E. grandis* genome v. 0.6 (http://phytozome.jgi.doe.gov/pz/portal.html#!info?alias=Org_Egrandis). The first 50 regions (hits) with over 85 % identity in a region over 250 bp were selected for full-length LTR-RTE screening. Regions 20 kb up- and downstream from these hits were analyzed using the LTR-Finder [21] and LTR_STRUC [22]. Only full-length LTR-RTEs that aligned with ESTs in BLASTN were retrieved for further analyses, and redundant sequences were discarded. These reference sequences were deposited at GenBank under accessions KM196471 to KM196479. Target site duplications (TSDs) were identified by submitting the full-length sequences as a query and subject to a blast2seq on NCBI website. Putative ORFs were retrieved using FGENESH + tool [23] on Softberry platform (http://linux1.softberry.com/berry.phtml) and manually inspected. Conserved domains were annotated using Pfam (http://pfam.xfam.org/). In the case of the two RLC_*egAngela* families, 5’ non coding leader sequences were compared using LALIGN (http://fasta.bioch.virginia.edu/fasta_www2/fasta_www.cgi?rm=lalign). RNA minimum free energy secondary structure of aligned regions (376 and 204 nt) was performed using RNAfold (http://rna.tbi.univie.ac.at/cgi-bin/RNAfold.cgi).

### Phylogenetic analysis and RTE family name assignment

All sequences from two previous large-scale analyses of plant LTR-RTEs [17, 24] were used to develop phylogenetic trees based on a reverse transcriptase fragment. We used a total of 95 *Copia* [17, 24] and 37 *Gypsy* [24] families to classify the nine *Eucalyptus* LTR-RTE families. DNA sequences were aligned using MUSCLE [25] with default parameters and the phylogenetic trees were made using MEGA 6.0 [26], applying the Maximum Likelihood method, with 1,000 bootstrap replicates. We used the Kimura-2-parameter substitution model and gap positions were excluded when present in more than 5 % of the sequences.

*Eucalyptus* LTR-RTEs were assigned to families within lineages on the basis of 80 % sequence identity in at least 80 % of their LTRs, based on the universal classification of TEs [1]. We standardized the name of *Eucalyptus* LTR-RTE sequences similar to Domingues et al. [6] rationale: they were named 'RLC' (*Copia*) or 'RLG' (*Gypsy*), 'eg' for '*Eucalyptus grandis*', the lineage name (e.g. '*Ale*') and the family number (e.g. '1').

### Theoretical protein modeling and molecular dynamics simulations

We selected amino acid sequence of RLG_*egTekay*_1 chromodomain, integrase and reverse transcriptase, as well as the integrase of RLC_*egAle*_1 and reverse transcriptase of RLC_*egBianca*_1 by manual selected translation of nucleotide sequences. The sequences were submitted to HHpred server [27], and further to MUSCLE [25] and MUSTER (MUlti-Sources ThreadER) [28]. The best alignments were used to generate models with Modeller v.9.12 [29]. The models were calculated with RAMPAGE [30] and ProSA-web [31] based on structural comparison with template. DNA/RNA molecules were added to the Integrases and Reverse Transcriptases theoretical models by superimposition of DNA binding region.

The best 13 models were submitted to molecular dynamics (MD) simulations using Groningen Machine for Chemical Simulation (GROMACS) v.4.5.3. [32]. The Charmm force field [33] was chosen with explicit solvent [34] and a minimum of 50 ns unrestrained simulation. Theoretical models stability was evaluated by average root mean square deviation (rmsd)/time graph and by overall stereochemical and energy quality. Figure and surface illustrations were generated in CHIMERA [35], with the electrostatic surface generated by APBS [36], PDB2PQR server (http://nbcr-222.ucsd.edu/pdb2pqr_2.0.0/) and PROPKA [37].

### Copy number determination in *E. grandis* genome

We used MEGABLAST to extract the full-length copies of the nine families from the *E. grandis* genome v 1.0 [13]. All matches that were at least 80 % of the length of the reference full-length sequences and had a similarity level higher than 80 % were considered for copy number analysis. Copies that did not harbor the canonical 5'TG..CA3' were manually removed. Complete copies were plotted in *E. grandis* genome using Circos [38].

### Estimation LTR-RTE insertion time and average LTR divergence sequence

The insertion time of intact LTR-RTE families with two complete LTR sequences and TSDs was calculated based on the assumption that they are identical at the time of integration [39]. For each element, we aligned 5’ and 3’ LTRs using the MUSCLE program implemented in MEGA 6.0 [26], with default parameters. Divergence between LTRs (K) was calculated using MEGA 6.0, using Kimura-2-parameter distance [40]. The insertion time (T) for each intact element was calculated with the formula: T = K/2r. The value of 1.5 x 10^-8^ substitution per site per year (r) [41] was recently used for the calculation of LTR-RTEs age in grape [42].

Average divergence (Pi) of LTRs for each LTR-RTE family was also calculated, using DnaSp program [43].

### *In silico* transcriptional analysis: *Eucalyptus* spp EST screening

LTR-RTEs full-length sequences were used as BLASTN queries against *Eucalyptus* ESTs from EUCANEXT database [44, 45], http://bioinfo03.ibi.unicamp.br/eucalyptusdb/). ESTs similar to LTR-RTEs were assigned to a family according to the criteria adapted from Wicker et al. [1]: 80 % coverage with 80 % nucleotide identity, in a region over 200 bp.

### Plant Material and nucleic acid extraction

For IRAP (Inter-Retrotransposon Amplified Polymorphism) and REMAP (Retrotransposon-Microsatellite Ampliflied Polymorphism) analyses, leaves were collected from 10 unrelated individuals of five *Eucalyptus* species: *Eucalyptus brassiana* S.T. Blake, *Eucalyptus grandis* W. Hill ex Maid., *Eucalyptus saligna* Sm., *Eucalyptus tereticornis* Sm. and *Eucalyptus urophylla* S.T. Blake. These individuals were maintained in field by Suzano Papel and Cellulose breeding program. Total DNA was extracted from fresh young leaves using the protocol described in Ferreira and Grattapaglia [46].

For all other analyses, *Eucalyptus* seedlings were grown under naturally fluctuating conditions of temperature and air relative humidity, and were fertilized and irrigated as necessary in a greenhouse from the Suzano Papel and Cellulose breeding program, in Itapetininga, São Paulo, Brazil. Throughout the experiment, the plants were randomized periodically to minimize any variation within light environment. All plants were harvested 90 days after seed planting.

In the relative quantification of LTR-RTE families, we used the total DNA of *E. grandis* (clone GD 33) *and E. urophylla* (clone URO11). In this case, genomic DNA was obtained from young leaves using the DNeasy plant kit (QIAGEN), as recommended by the manufacturer.

For RNA extraction we used freshly harvested leaves, stalk and secondary roots from the five *Eucalyptus* species mentioned above and one hybrid *E. grandis* x *E. urophylla* (termed “E. urograndis”). For each tissue, total RNA was extracted from two groups composed by five plants each according to modificated CTAB protocol proposed by Korimbocus et al. [47]. RNA integrity was checked by electrophoresis, in denaturing agarose gel. RNA quality and quantification was analyzed by spectrophotometry at 260 nm and 280 nm (NanoDrop ND-1000, Thermo Scientific) and stored at -80 °C until used.

### IRAP and REMAP profile and data analysis

Sixteen single IRAP primers were designed based on nine LTR-RTE families of *E. grandis* genome v. 1. (Additional file 1: Table S1). The procedures of PCR amplification were adapted from the protocol of Smýkal [48]. Reactions were done in a total volume of 10 μl, containing 25 ng of genomic DNA, 0.7 X PCR buffer (750 mM Tris-HCl, 200 mM (NH_4_)_2_SO_4_), 25 mM MgCl_2_, 40 ng of *primer*, 0.4 mM of each dNTP and 0.3 U of *Taq* DNA polymerase (Fermentas). The amplification profile consisted of an initial denaturation at 94 °C for 4 min, followed by 35 cycles at 94 °C for 40 s, 50 °C for 2 min, 72 °C for 3 min and 50 s and a final extension of 5 min at 72 °C.

For REMAP, we used the combination of sixteen IRAP primers with 10 SSR primers described in Kalendar et al. [49] (Additional file 1: Table S1). PCR reactions contained 40 ng of genomic DNA, 1.0 X PCR buffer (750 mM Tris-HCl, 200 mM (NH_4_)_2_SO_4_), 25 mM MgCl_2_, 10 ng of each *primer*, 0.6 mM of each dNTP, and 0.3 U of *Taq* DNA polymerase (Fermentas). The amplification profile composed of an initial denaturation at 94 °C for 4 min, followed by 35 cycles at 94 °C for 1 min, 56 °C for 1 min, 72 °C for 1 min and 50 s, and a final extension of 5 min at 72 °C. All IRAP and REMAP reactions were carried out in a PTC-100 thermocycler (MJ Research, Inc.) and PCR products were resolved in 1.8 % agarose gels stained with ethidium bromide.

Each IRAP and REMAP band was treated as a single locus. The presence or absence of a given fragment length in each sample was recorded manually in binary code. DNA marker data was processed by NTSYS-pc version 2.10 [50] and using the SIMQUAL module with the Jaccard genetic similarity coefficient (GSj), and the similarity data was used to perform an unweighted pair group method with arithmetic mean (UPGMA) cluster analysis using the SHAN module, following Fan et al. [51].

### LTR-RTE quantification in *Eucalyptus urophylla*: quantitative real time PCR

Eight LTR-RTE families from *E. grandis* were quantified in *E. urophylla* using quantitative real time PCR (qPCR) using the method of Kraitshtein et al. [52] and Baruch and Kashkush [53], which is based on a comparative 2^−ΔΔCt^ method, using a single-copy gene as a reference. Our reference gene was DUR3, a urea transporter, which is a single-copy gene in several eukaryotes [54, 55], and in *E. grandis* genome we found only a single copy in chromosome 5 (data not shown).

Primers were designed using PerlPrimer v1.1.17 software (http://perlprimer.sourceforge.net) in LTR and internal regions (Additional file 1: Table S1). In order to confirm target specificity we cloned and sequenced amplified regions (ABI 3130xl, Applied Biosystems). Quantitative PCR melt curves also revealed single and unique peaks for each primer pair, confirming high specificity to the target sequence fragments.

PCR efficiencies of the target and reference genes were determined by generating standard curves, based on serial dilutions prepared from cloned DNA templates. We made serial dilutions of the control DNA from 5x10^-1^ to 5x10^-6^ ng/μl, with 0.15 ng of each primer.

Fold amplification in each cycle was calculated according to PCR efficiency, which was deduced by the software from the slope of the regression line (y) according to the equation E = [(10 − 1/y) − 1] × 100. For primers with 100 % efficiency, the fold equals 2.

qPCR reactions were conducted in a Step One Plus Real Time PCR System (Applied Biosystems) and analyzed in Step One 2.1 software (Applied Biosystems).

Each qPCR reaction was performed in 5 μl of GoTaq® qPCR Master Mix (Promega), with 1.0 ng of each primer and 3.7 μl of ultra-pure water. The cycling conditions were as follows: 5 min at 95 °C, followed by 45 cycles each of 15 s at 95 °C, 60 s at 60 °C. In order to confirm the reproducibility of our results, reactions were done in technical triplicates in three independent experiments using 0.125, 0.25 and 0.5 ng of genomic DNA.

The relative quantities of LTRs and internal regions of *Copia* and *Gypsy*-like LTR-RTEs families were calculated according to Kraitshtein et al. [52] and Baruch and Kashkush [53].

### LTR-RTE Transcriptional analysis by RT-qPCR

In addition to plant material mentioned above, in RT-qPCR analysis, we also evaluated the transcriptional impact of osmotic shock in secondary roots of *E. grandis* submerged in hydroponic solution with PEG. Plants were harvested 0, 6 and 24 h after osmotic stress. Additional details of this experiment are detailed in Rodrigues et al. [56].

All RNA samples were treated with DNase I (Fermentas) and reverse transcribed using GoScript™ Reverse Transcription System kit (Promega). RT-qPCR reactions were performed in technical triplicates from at least two biological replicates in a StepOnePlus Real Time PCR System (Applied Biosystems). Primers used for RT-qPCR are described in Additional file 1: Table S1, and fragments were cloned and sequenced confirming target amplification. Each biological replicate was represented by bulks of leaves, stalk and secondary roots from five plants.

Reactions contained 5 μl of 2 x mix GoTaq® Master Mix (Promega), 1.0 ng of each primer, 150 to 280 ng of cDNA and ultra-pure water up to a final volume of 10 μl. The cycling conditions were as follows: 5 min at 95 °C, followed by 45 cycles each of 15 s at 95 °C, 60 s at 60 °C. Relative expression levels were normalized using the GAPDH gene [57]. The GAPDH stability was evaluated using geNorm and NormFinder. Expression stability was <0.15 in geNorm and <0.03 in NormFinder for all species and tissues (data not shown). These values were compatible with the most stable normalizers from a previous publication in *Eucalyptus* [57]. In geNorm, stability values below 0.15 do not require an additional gene as a reference [58, 59].

The tissue/organ with lowest expression (highest Ct) was used as calibrator (expression value = 1).

RT-qPCR efficiency was calculated using Linreg v. 2013.0 [60], and reactions with efficiency >90 % were used for analysis. Relative expression was calculated using ΔΔCt method [61] with the formula (1 + E)^ΔΔCt^, where E represents the efficiency. The statistical analysis was performed using Assistat 7.7 beta [62]. We used one-way analysis of variance (ANOVA) and in cases where significant differences were found, the Least Square Deviation (LSD) method for multiple comparisons was performed. Results were considered significant at P < 0.05.

## Results

### LTR-retrotransposons in *Eucalyptus*: overall view and phylogenetic structure

**Fig. 1:**
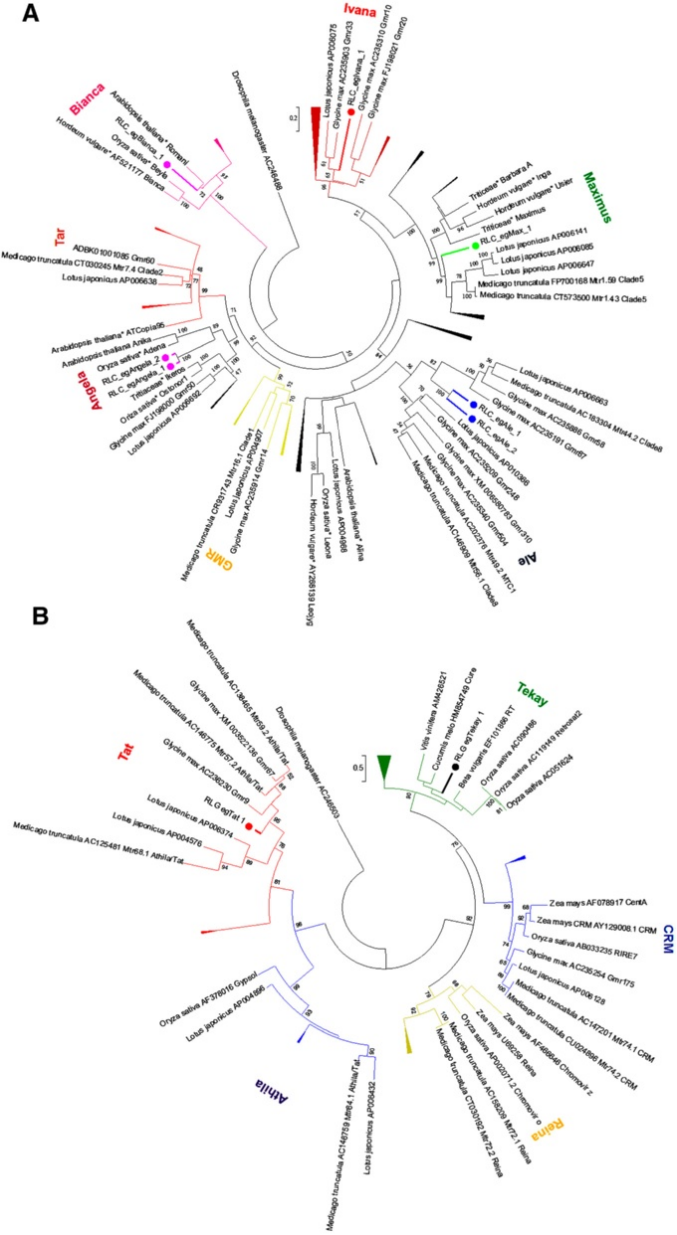
Domains in *Eucalyptus* LTR-RTEs. **a** *Copia* superfamily; **b** *Gypsy* superfamily. Abbreviations and domain color coding: LTR = long terminal repeat (pink); PBS = protein binding site and PPT = poly-purine tract (dark blue); Gag (blue); RNAseH = Ribonuclease H (light blue); integrase = Integrase (purple)

By using a homology-based approach, we mined a total of nine transcriptionally active LTR-RTE families in the *E. grandis* genome. Seven families belong to the *Copia* superfamily and two correspond to LTR-RTE elements from the *Gypsy* superfamily. *Copia* superfamily LTR-RTEs were classified into five major plant evolutionary lineages [17] (Fig. 1a) and two *Gypsy* lineages [24] (Fig. 1b). We did not found any *Copia* element from *GMR* and *Tar* lineages. Three *Gypsy* lineages - *CRM*, *Reina* and *Athila* - did not harboured any transcriptionally active LTR-RTE in our analysis.

**Fig. 2:**
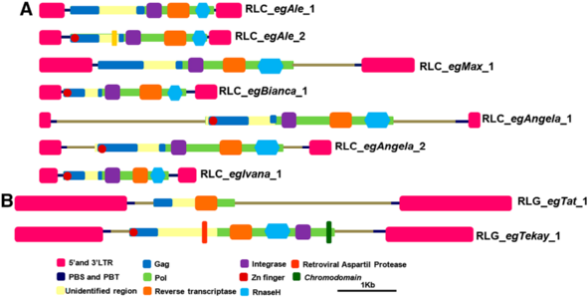
Classification of *Eucalyptus grandis* LTR-RTEs *Copia* and *Gypsy* superfamily sequences into nine new families. Phylogenetic analyses used 95 *Copia* sequences from Wicker et al. [1] and 37 *Gypsy* sequences from Du et al. [24] **a** Evolutionary lineages of seven elements from *Copia* superfamily in *Eucalyptus* **b** Evolutionary lineages of two Eucalyptus *Gypsy* LTR-RTEs

The reference full-length sequences of these families (Table 1) have between 93 and 99 % identity among 5’and 3’LTRs, and for all reference sequences we annotated at least two internal coding domains among *gag*, reverse transcriptase, integrase and RNAseH (Fig. 2). RLC_*egAngela*_1 and RLC_*egAngela*_2 families have > 95 % identity between internal coding domains; however, LTRs have < 80 % similarity; consequently, we preferred to classify these elements as separate families. We also compared 5’ non coding region in order to confirm if they correspond to distinct families, similar to Tanskanen et al. [63]. The folding pattern of 5’ leader region was completely distinct between the two families (Additional file 2: Figure S1), confirming that they represent two different families.

**Table 1:** Overall features of LTR-RTEs identified in *E. grandis* genome

Superfamilies	Families	Lineages	LTR length (bp)	LTRs Id^a^	LTR-RTE size (bp)	Copy number
*Copia*	RLC_*egAle*_1	*Ale*	458/457	99 %	5509	12
	RLC_*egAle*_2	*Ale*	434/434	96 %	5395	4
	RLC_*egMax*_1	*Maximus*	904/903	98 %	9670	623
	RLC_*egBianca*_1	*Bianca*	424/423	99 %	5008	77
	RLC_*egAngela*_1	*Angela*	162/161	93 %	11280	1
	RLC_*egAngela*_2	*Angela*	392/392	92 %	7473	67
	RLC_*egIvana*_1	*Ivana*	247/239	93 %	4440	49
*Gypsy*	RLG_*egTat*_1	*Tat*	2199/2257	93 %	18300	15
	RLG_*egTekay*_1	*Tekay*	555/552	96 %	12159	7

^a^Id: identity among 5’ and 3’LTRs

Overall size followed previous large-scale reports for LTR-RTEs, as well for PBS and PPT sequences (Additional file 1: Table S2). Both *Gypsy* families have > 12 kbp and the average size of *Copia* is around 7 kpb (Table 1). LTRs from *Gypsy* elements are also larger: only RLC_*egMax*_1 has LTRs longer than 500 bp (Table 1). Five families contained a spacer region between internal coding domain and LTRs (Fig. 1).

### Structural characterization and molecular dynamics simulations of LTR retrotransposon domains

**Fig. 3:**
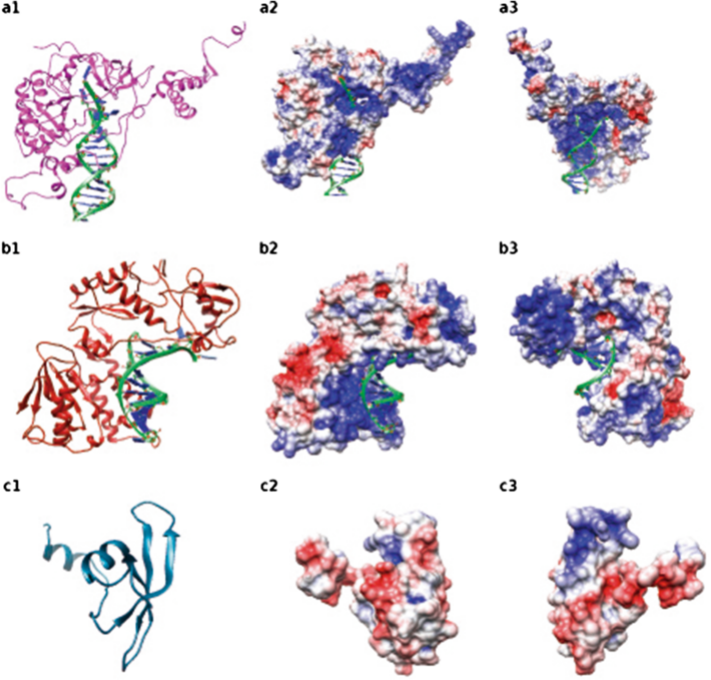
Three dimension theoretical models of RLG_*egTekay*_1: Integrase (INT1) (**a**), Reverse Transcriptase (RT1) (**b**) domains complexed with DNA and Chromodomain (**c**). In 1, protein cartoon representation. In 2 and 3, electrostatic surface in red (-4) acid regions and in blue (4) basic regions rotated in 180°

**Fig. 4:**
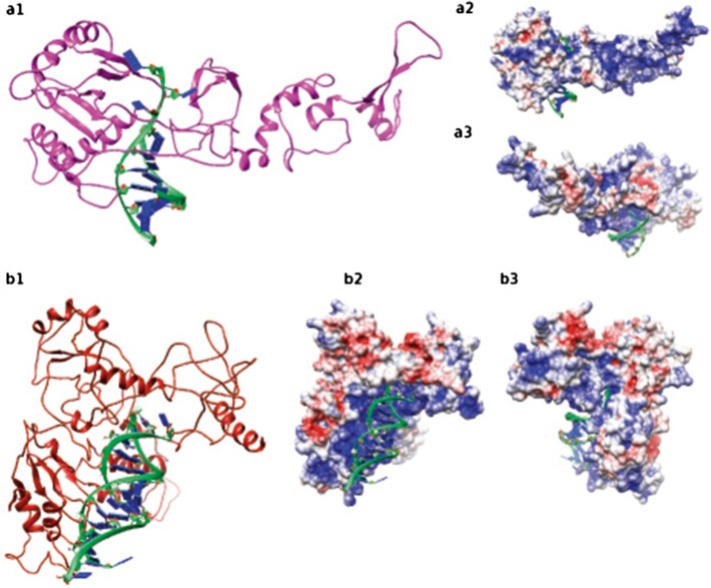
Three dimension theoretical models of Integrase (INT2) of RLC_*egAle*_1 (**a**) and Reverse Transcriptase (RT2) (**b**) of RLC_*egBianca*_1 proteins complexed with DNA. In 1, protein cartoon representation. In 2 and 3, electrostatic surface in red (-4) acid regions and in blue (4) blue regions rotated in 180°

By threading modeling, we generated the first theoretical models of LTR-RTE proteins in plants. We used three domains of RLG_*egTekay*_1: chromodomain (CD1) (Fig. 3a), integrase (INT1) (Fig. 3b) and reverse transcriptase (RT1) (Fig. 3c). We also generated domains theoretical models on the domains integrase of RLC_*egAle*_1 (INT2) (Fig. 4a) and reverse transcriptase of RLC_*egBianca*_1 (RT2) (Fig. 4b). The template used to generate CD1 was the NMR structure of a chromodomain (PDB id/monomer: 2RSO/A) from *Schizosaccharomyces pombe* [64]. The template used to generate INT1 was the crystallographic structure of an inhibited retroviral integrase (3OYM/A) from *human Spumaretrovirus* [65] and M-phase phosphoprotein 8 (PDB id: 3LWE/A) from *Homo sapiens* [66]. The template used to generate RT1 was the crystallographic structure of a reverse transcriptase/ribonuclease H p80 complexed with RNA/DNA hybrid (4HKQ/A) from *Schizosaccharomyces pombe* [67]. The template used to generate INT2 was the crystallographic structure of PFV integrase (3OYM/A) from *Human spumaretrovirus* [65]. The template used to generate RT2 was the crystallographic structure of Ty3 reverse transcriptase complexed to RNA/DNA hybrid (4OL8/A) from *Saccharomyces cerevisiae* [68]. The CD1, INT1, RT1, INT2 and RT2 had original models with 93.6 %, 98.3 %, 98.5 %, 94.2 % and 96.8 % of residues in favored and allowed region which were improved to 100 %, 99.7 %, 99.3 % 98.2 % and 98.7 %, respectively, after MD dynamics. These models started with a Z-score of -0.87, -5.49, -9.7, -4.41 and -3.82 which was either improved or maintained to -1.03, -5.41, -8.91, -5.04 and -4.02. All of the models were submitted to 50 ns and had its stabilization within the first 30 ns with a rmsd of 0.35 to 1.25 nm. Thus, the generated models were stabilized by long MD simulations and validated by different approaches.

**Fig. 5:**
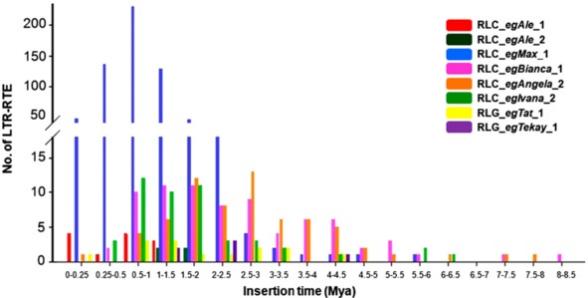
Estimative insertion time of LTR-RTE families in *Eucalyptus grandis* genome

### Quantification of LTR-RTEs

A total of 843 full-length elements from *Copia* superfamily and 22 from *Gypsy* superfamily were identified, and copy number per family ranged from 1 to 623 (Table 1). The distribution of each family is distinct among *E. grandis* chromosomes when compared to gene and repeats density (Additional file 2: Figure S2). RLC_*egMax*_1 is the most ubiquitous, in accordance with the prevalence of *Maximus* LTR-RTE families in plants. This family is dispersed along the chromosome arms in both gene-rich and repetitive-rich regions (Additional file 2: Figure S2c) without any specific preference. Full-length RLC_*egMax*_1 copies correspond to ~1 % of *E. grandis* genome. RLC_*egBianca*_1 and RLC_*egAngela*_2 families presented a similar distribution, preferentially inserted in repeat-rich regions (Additional file 2: Figure S2d,f). RLC_e*gAngela*_1 have only one copy in chromosome 3, also located in a repeat-rich region (Additional file 2: Figure S3e). Both LTR-RTEs from *Gypsy* superfamily also have an insertional preference over repeat-rich regions (Additional file 2: Figure S2j,k). On the other hand, RLC_*egIvana*_1 was preferentially inserted in gene-rich regions of chromosomes 2, 3, 7 and 9 (Additional file 2: Figure S2g).

Additionally, we also quantified LTRs and internal regions of families by qPCR in *E. urophylla* using *E. grandis* as a calibrator. *E. urophylla* is among the most commonly used species in the paper industry in Brazil and belongs to the same subgenus of *E. grandis* [69, 45].

**Fig. 6:**
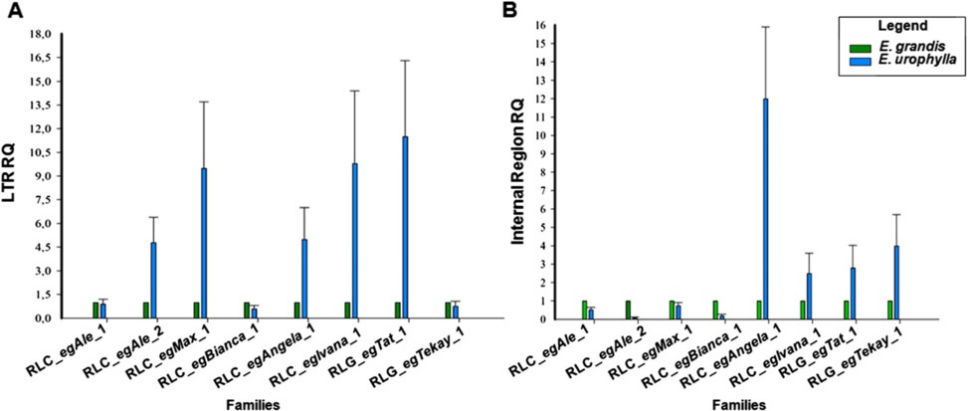
Copy number of *Copia* and *Gypsy* LTR-RTE families *E. grandis* and *E. urophylla* using qPCR. **a** RQ of LTRs; **b** RQ of internal regions

*In silico* quantification and qPCR had similar results, except for RLC_*egAle*_2 which qPCR analyses showed more copies than *in silico* analyses. The proportion of LTRs for four retrotransposons families, RLC_*egAle*_1, RLC_*egMax*_1, RLC_*egIvana*_1 and RLG_*Tat*_1, is higher in *E. urophylla* than *E. grandis* (Fig. 6a). This pattern is different in internal regions, where only two, RLC_*egAngela*_1 and RLC_*egTekay*_1, have a higher copy number in *E. urophylla*, suggesting a diversification in LTR regions. RLC_*egAngela*_1 and RLC_*egAngela*_2 families display the most prominent difference in coding regions, increasing significantly in *E. urophylla* compared to *E. grandis* (Fig. 6b).

### Insertion time of intact elements

We estimated the insertion time of all LTR-RTE families that harbor at least five intact copies. The 12 RLC_*egAle*_1 copies were inserted into the genome <1.5 Mya, whereas most RLC_*egMax*_1 copies (98.38 %) were inserted into the genome between 0 – 2.5 Mya (Fig. 5). RLC_*egBianca*_1 and RLC_*egAngela*_2 copies have a similar pattern of insertion time where most of the copies were inserted into the genome 0.5 – 4.5 Mya. Approximately one third of the copies from RLC_*egBianca*_1 (24) and RLC_*egAngela*_2 (23) insertion has >3.0 Mya, including two copies dated to >7.5 Mya in both families (Fig. 5). The majority of RLC_*egIvana*_1 copies were inserted in the genome between 0.25 - 2.0 Mya (Fig. 5). The two LTR-RTEs from *Gypsy* superfamily have distinct patterns: while RLG_*egTat*_1 have most of these copies inserted 0.5 – 3.5 Mya (Fig. 5), RLG_*egTekay*_1 has a uniform distribution. We found one copy with two identical LTRs, in RLC_*egAngela*_2, five identical LTRs in RLC_*egMax*_1. The diversity (Pi) of LTRs ranged from 0.25 (±0.00379) (RLC_*egBianca*_1) to 0.35 (±0.17742) (RLC_*egAngela*_1) (Additional file 1: Table S3) and both *Gypsy* families have an average diversity of 0.31 (±0.023 from RLG_*egTat*_1 and ± 0.045 from RLG_*egTekay*_1).

### IRAP and REMAP polymorphisms within *Eucalyptus* genus

Fifty *Eucalyptus* spp unrelated individuals were scored by IRAP and REMAP, yielding in total 3096 fragments, among which 700 were polymorphic. IRAP bands ranged from 250 bp to 2.25 kb (Additional file 2: Figure S3) and REMAP fragments ranged from 50 bp to 1 kb, except for primers from RLC_*egAle*_2 family (Additional file 2: Figure S4). Fifteen single IRAP primers and 23 REMAP primer combinations yielded reliable results. The number of scorable bands per primer in IRAP ranged from 18 (*E. urophylla*; 2.4 % polymorphic) to 20.8 (*E. saligna*; 4.6 % polymorphic). For REMAP bands ranged from 11.9 (*E. brassiana*; 4.3 % polymorphic) to 15.6 (*E. urophylla*; 2.1 % polymorphic) (Additional file 1: Table S3 and S4).

**Fig. 7:**
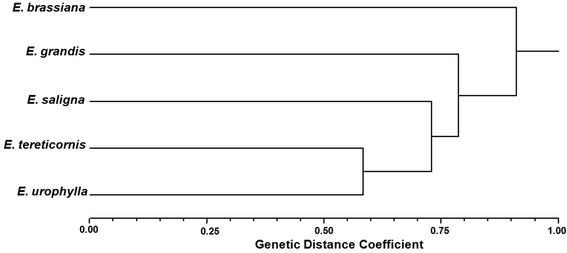
UPGMA dendrogram of five *Eucalyptus* species using IRAP and REMAP data based on Jaccard distance

The genetic relationships of these genotypes were unrevealed using the UPGMA method based on Jaccard (ranging from 0 to 0.92) computed with IRAP and REMAP markers (Fig. 7, Additional file 2: Figure S5). *E. brassiana* remained an outgroup, and the most related species were *E. tereticornis* and *E. urophylla. E. grandis* was the second most external species, after *E. brassiana*.

### Transcriptional activity of LTR retrotransposons families

In order to further characterize the transcriptional profile of LTR-RTE families, we performed BLAST searches against EST of six *Eucalyptus* species available in the EUCANEXT database [44, 45]. More than 150 ESTs matched the selected LTR-RTE families, and results are shown in Additional file 1: Table S3. RLC_*egBianca*_1 and RLG_*egTat*_1 were the most represented families (Additional file 1: Table S3). RLC_*egAle*_1, RLC_*egAle*_2, and RLG_*egTekay*_1 showed similarity with ESTs from three of six analyzed *Eucalyptus* species (Additional file 1: Table S3), and one EST of *E. globulus* displayed high similarity RLC_*egIvana*_1 (Additional file 1: Table S3). RLC_*egMax*_1 only had similarity with expressed sequences from *E. globulus*, (Additional file 1: Table S3).

**Fig. 8:**
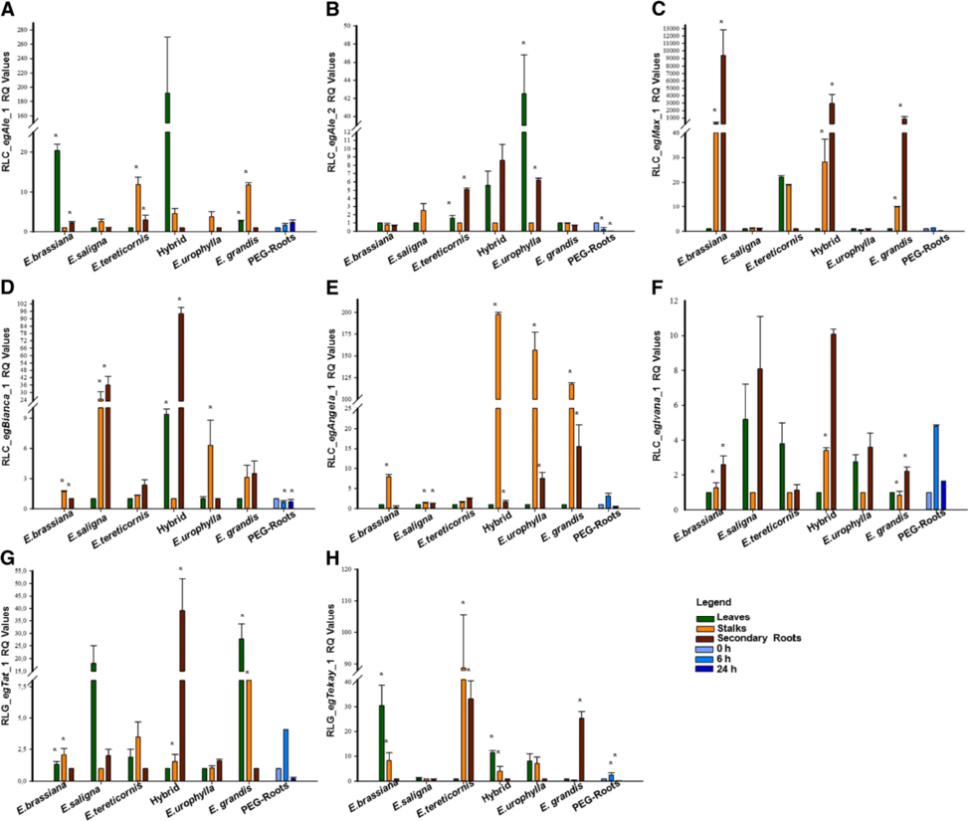
Transcriptional profile of *Eucalyptus* LTR-RTEs in three organs (detailed in panel "Legend") from five *Eucalyptus* species and one interspecific hybrid using RT-qPCR. Asterisk indicating standard error (n = 9) in bars (*p ≤ 0.05, ANOVA followed by LSD test)

A second approach, RT-qPCR of coding regions, was employed to detect transcriptional levels of LTR-RTE families in three tissues (leaves, stalk and secondary roots) from five *Eucalyptus* species (*E. brassiana*, *E. grandis*, *E. saligna*, *E. tereticornis* and *E. urophylla*) and one hybrid (*E. grandis* x *E. urophylla* – termed “E. urograndis” to facilitate discussion). *E. grandis* secondary roots were also evaluated in osmotic stress imposed by PEG treatment [61] (Fig. 8).

This analysis expanded and detailed EST analysis, confirming that families have differential transcriptional activity *Eucalyptus* spp tissues. RLC_*egAle*_1 highest transcriptional levels were in leaves from “E. urograndis” and *E. brassiana*, with a remarkable level in stalks of *E. tereticornis* and *E. grandis* (Fig. 8a). Regarding the RLC_*egAle*_2 family, leaves from *E. urophylla* presented the highest transcriptional activity (Fig. 8b). RLC_*egMax*_1 family showed a high level of transcriptional activity in secondary roots from *E. brassiana* and *E. grandis*, and in “E. urograndis” we observed a notable difference compared to parentals *E. grandis* and *E. urophylla* (Fig. 8c). RLC_*egBianca*_1 family also displayed a higher transcriptional level in secondary roots of “E. urograndis”, and it was the most expressed family in stalks and roots of *E. brassiana* (Fig. 8d). LTR-RTEs of the *Angela* lineage (primers could not distinguish families) have a remarkable level in stalks in three *Eucalyptus* species and “E. urograndis”, and a low transcriptional level in leaves of all *Eucalyptus* species studied (Fig. 8e). The RLC_*egIvana*_1 family had a ubiquitous expression level (Fig. 8f).

*Gypsy* elements showed a distinct transcriptional level according to the family, tissue and *Eucalyptus* species evaluated. RLG_*egTat*_1 showed a remarkable expression in leaves of two *Eucalyptus* species (see Fig. 8g), and in stalks from *E. saligna* and secondary roots from “E. urograndis” (Fig. 8g). On the other hand, RLG_*egTekay* _1 family showed the highest transcriptional activity in stalks from *E. tereticornis* (Fig. 8h).

The transcriptional level of LTR-RTEs in roots submitted to osmotic stress by PEG treatment increased after 6 h in five families (RLC_eg*Ale*_1, RLC_eg*Max*_1, RLC_eg*Bianca*_1, RLC_eg*Ivana*_1, RLG_eg*Tat*_1 and RLG_eg*Tekay*_1) and decreased after 24 h (Fig. 8). Only in the RLC_*egAle*_1 family did we observe a higher expression after 24 h (Fig. 8). RLC_*egAle*_2 and RLC_*egBianca*_1 families showed a reduction in transcription level following PEG application (Fig. 8).

## Discussion

### Novel LTR-RTE families in *Eucalyptus* genus have individual molecular patterns

This study represents a fine-scale analysis of transcriptionally active LTR-RTEs in *Eucalyptus* species, taking advantage of the recently released *E. grandis* genome and expanding *in silico* analyses to a comparative study in terms of copy number, polymorphism insertion and tissue-specific transcriptional pattern.

The majority of the identified LTR-RTE families were from the *Copia* superfamily, consistent with previous EST analysis of LTR-RTEs in sugarcane [6] and coffee trees [7] but in contrast to wheat [70] and maize [5]. These findings confirm that the superfamily transcriptional preference is variable among plant genomes.

Most families from the same evolutionary lineage have similar size, LTR length and high similarity between PPT and PBS regions with previous multi-scale reports [17, 24]. Three *Copia* families displayed a spacer region between ORF and LTRs: RLC_*egMax*_1, with a 1 kb spacer region at 3-UTR. The RLC_*egAngela*_1 and RLC_*egAngela*_2 has a spacer region of 5580 pb and 3205 pb at 5-UTR, respectively. Despite the high identity between internal coding domains between *Angela* families, their LTRs display differences in size and similarity; moreover, folding analyses of the 5’UTR also support that they represent distinct families (Additional file 2: Figure S1). RLC_*egAngela*_1 has a larger spacer region between LTRs and this region has 99 % identity with an *Eucalyptus* spp EST (GenBank accession HS066626, data not shown), suggesting that spacer regions are also transcriptionally active. RLC_*egAngela*_1 and RLC_*egAngela*_2 have strikingly high similarity in coding regions, but they habour dissimilar LTR and untranslated regions. This feature is particularly common in *Copia* superfamily, (i.e, BARE1 and 2) [63]. We also discovered that one transcriptionally active LTR-RTE family initially classified as a member of the *Gypsy* superfamily are in fact caulimoviruses, with 58 complete copies in the *E. grandis* genome with EST support (data not shown). Previous studies in plants provided similar results [15], and the identification of ESTs matching plant caulimoviruses suggest that these elements may have a significant impact in transcriptome of angiosperms.

*Gypsy* families can be roughly divided according to the presence of a *Chromodomain* in the C-terminal of the integrase domain. RLG_*egTekay*_1 family is a typical *Chromoviridae* member, closely related to the sugarbeet element *Bongo3* [71]. But, differing from *Bongo3*, a high-copy element spread throughout all sugarbeet chromosomes, only 5 *E. grandis* chromosomes have full-length RLG_*egTekay*_1 copies.

### *Eucalyptus* LTR-RTE proteins interact with DNA and RNA

Up to now, few studies address TEs molecular structure [72–74] and here we deliver the first study to theoretically model and stabilize with long MD simulations domains of LTR-RTE proteins in plants. The generated models posses a wide distribution of unordered regions, above all the *Copia* models (Fig. 4) [72]. Comparison of integrase models (Figs. 3a1 and 4a1 representation colored in pink) clearly show variability between members (rmsd of 7.2 Å), although superimposition of DNA interacting region yields lower value (3.17 Å). The same is observed in reverse transcriptases (Figs. 3b1 and 4b1 representation colored in dark red) with lower values, which shows higher structural conservancy. The high rmsd values are due to the flexibility between cores given by connecting loops and unordered regions, although the cores are similar explaining the conservancy of function. The variance between models corroborates the differences observed in previous sequence analysis, since RLC_*egAle*_1 and RLC_*egBianca*_1 are from different families and the RLG_*egTekay*_1 from a different superfamily. The electrostatic surface of Integrase and Reverse Transcriptase show the complementarities region with RNA/DNA (Figs. 3a3–b2, 4a3–b2). The Chromodomain was not modeled with RNA since its interaction region is not fully understood. This model consists in its general structure, an N-terminal three-stranded anti-parallel β-sheet (β-sandwich) packed against C-terminal α-helix (Fig. 3c1) and it has mostly acid regions. Further studies should be performed to aid transposons domains structural comprehension, such as modeling the whole ORFs from the LTR-RTE.

### Most LTR-RTE families are recently inserted in *Eucalyptusgrandis *genome

Our analysis highlights the fact that even in transcriptionally active LTR-RTEs we could track contrasting insertion peaks. We dated the most part of full-length LTR-RTEs insertions dated <3 Mya (Fig. 5). This characteristic is similar to other plants, such as rice [75], *Vitis* [76] and tomato [77]. The identification of insertions >3Mya old is difficult in plant genomes [78, 77], because old LTR-RTEs insertions could be removed by recombination processes, as well as mutational events on nucleotide composition.

Six out of 8 analyzed families showed recently inserted copies (<1.0 Mya), with RLC_*egAngela*_2 and RLC_*egMax*_1 families having copies with estimated insertion of <0.1 Mya. RLC_*egMax*_1, which represents ~1 % of *E. grandis* genome, has a peak of amplification between 0.5 and 1.5 Mya. Most insertions over 4.5 Mya correspond to elements from 2 families: RLC_*egBianca*_*1* and RLC_*egAngela*_2, suggesting that forces driving RLC_*egAngela*_2 amplification are evolutionarily old and still in action. This is consistent with analyses in other plants genomes that insertion >6 Mya, such as *Populus tricocarpa* [79], onion and asparagus [80].

LTR-RTEs in plants can be either localized in gene-rich regions [81, 77] or in repeat-rich regions [82], which also represents the scenario of *Eucalyptus* retrotransposons here characterized. Although chromosome 3, 5 and 8 concentrated most of annotated full-length copies, *Eucalyptus* LTR-RTE families had distinct copy distributions, both in genic and repetitive regions. The most part of younger (<3Mya) LTR-RTEs inserted in genic regions, similar to the gene-rich euchromatin distribution from SALIRE family in *Beta vulgaris* [83] and LTR-RTEs in tomato genome [74]. Few full length copies of RLC_*egIvana*_1 (5), RLC_*egBianca*_1 (2), RLC_*egAngela*_2 (1) and RLC_*egMax*_1 (9) are inserted near to telomeric regions, suggesting that these families are not directly related to telomeric repeats.

The quantification of LTR-RTE families by qPCR has been performed in several species [84, 53, 16] and we successfully used this strategy to compare amplification of *Copia* and *Gypsy* elements in *E. grandis* and *E. urophylla*. qPCR quantification did not follow *in silico* quantification just for RLC_*egAle*_2 which qPCR analyses showed more copies than *in silico* analyses. This fact may be due to the *E. grandis* genotype used in qPCR analyses (Clone GD 33) that is not the same from genome sequencing (BRASUZ1), and defective LTR-RTEs may be overrepresented in *E. grandis* genotype here sampled.

This approach also helped us to track out the proportion of non-autonomous LTRs and internal regions. *E. grandis* (1C = 630 Mb) and *E. urophylla* (1C = 640 Mb) genomes have similar size and diverged <20 Mya [13] enabling the comparison of LTR-RTE families shared by both genomes.

RLC_*egAle*_1 have similar LTR and internal domain proportions for both genomes, suggesting that this family have not gone through an expansion burst since *E. grandis* and *E. urophylla* divergence. RLC_*egAle*_2, RLC_*egMax*_1, RLC_*egIvana*_1 and RLG_*egTat*_1 families had an increase of LTRs compared to internal domains in *E. urophylla*, which may indicate the propagation of LTRs, including non-autonomous elements, in this genome and may also reflect a fast substitution processes among internal domains of these families. RLC_*egAngela*_1 and RLG_*egTekay*_1 had the opposite profile, with a higher proportion of internal domains. This observation may indicate a higher divergence of LTRs that were not recognized by the primer combination used in our approach and conservation in coding domains, similar to the pattern of the *Angela* family within the *E. grandis* genome.

Three LTR-RTEs families, RLC_*egAle*_1, RLC_*egMax*_1 and RLG_*egTat*_1, showed an approximate proportion of two LTRs to each element. These families have most of their copies have young insertions, indicating that they probably did not harboured recombination processes. The qPCR analyses from RLC_*egAle*_2, RLC_*egAngela*_1 and RLC_*egBianca*_1 families showed more copies of internal regions than LTRs, which suggest a loss of LTR and internal region conservation in *E. grandis* genomes.

### IRAP and REMAP markers suggest distinct activities of LTR-RTEs families in *Eucalyptus* species

IRAP and REMAP markers may contribute to understanding the insertion activity of LTR-RTE families in *Eucalyptus* species. Most primer combinations were successfully applied in *Eucalyptus* species, showing the ubiquity character of families among the *Eucalyptus* genus. *E. grandis* showed more fragments and polymorphic bands, indicating that LTR-RTEs families had distinct activity after speciation events in this specie. *Copia* LTR-RTEs studied in four species from *Vitis* genus had a similar pattern, with polymorphic bands suggesting an amplification burst after speciation events [76].

The average size of REMAP fragments was probably the result of proximity between LTR and SSR regions than LTR-RTEs in tandem insertions. The pattern of REMAP fragments per *Eucalyptus* species reflect the preferential insertion events of LTR-RTE families in SSR regions with repetition motifs [(CT)_10_G] and [(AG)_10_ T], probably sampling LTR-RTEs located in pericentromeric regions, which are gene-poor and enriched in repetitive sequences, especially retrotransposons [85]. The high level of polymorphisms suggests that LTR-RTEs are extensively heterogeneous among *Eucalyptus* species, as observed in *Diospyros* sp. and *Medicago sativa* [24, 86].

This is the first report using IRAP and REMAP markers for genetic diversity in *Eucalyptus*, and genomic polymorphism suggests differential activity among RTEs within subgenus *Symphyomyrtus*. Those species occupy the same clade within subgenus *Symphyomyrtus* [87] but it is important to notice that the separation between those *Eucalyptus* species was not completely supported by bootstrap analyses. Distribution of *Eucalyptus* species in dendrogram has some differences comparing to molecular analyses based on DArT markers [87]. *E. tereticornis* and *E. urophylla* were the most close in RTE-based tree differently from the close relation usually present between *E. grandis* and *E. urophylla*, also observed for *E. brassiana* and *E. tereticornis* using other nuclear markers. On the other hand, our analysis shows a small distance between *E. saligna* and *E. grandis*, in agreement with a previous report using a nuclear gene [88].

### Transcriptional activity of *Eucalyptus* LTR-RTE families is variable among organs and species

The annotation of ESTs related to LTR-RTEs was an initial assessment of transcriptional activity of these elements in *Eucalyptus* genomes. RLC_*egAle*_1 was the family with the largest number of ESTs. RLC_*egBianca*_1 was the most ubiquitous element, with EST in the six mined *Eucalyptus* species. Detailed information of ESTs matching LTR-RTEs is available in Supporting Information Additional file 1: Table S6.

RT-qPCR clearly demonstrates that families have differences of transcriptional activity among *Eucalyptus* spp. tissues and species (Fig. 8). LTR-RTEs copy number have been suggested a cause to transcriptional increase, because more copies inserted in gene-rich euchromatin region [77] and probably near genes. Nevertheless, our data showed that no relation between LTR-RTEs copy number and expression level increase.

The transcriptional activity of families with the highest copy number, RLC_*egMax*_1, RLC_*egBianca*_1 and RLC_*egIvana*_1, was higher in secondary roots when compared to leaves. On the other hand, families with lower genomic copies are highly expressed in stalks and lower expressed in roots, i.e, RLC_*egAle*_1, RLC_*egAle*_2 and RLC_*egAngela*_1.

RLC_*egAle*_1 and RLC_*egAle*_2 families, despite their similar structure, have remarkable differences in their transcriptional patterns. The most striking differences can be observed in *E. brassiana*, *E. urophylla* and “E. urograndis” leaves, where each element displayed a species-specific transcriptional pattern. LTR-RTEs activation in hybrids was also described in other eudicots, like sunflower [84, 89] and tobacco [90].The activation of LTR-RTEs in hybrids may reflect two issues: an organ-specific deregulation of transcription factors that target LTRs in “urograndis” and/or a specific deregulation of silencing mechanisms regulating TE transcription.

The expression levels from RLC_*egAle*_1 in roots were lower to other tissues in all *Eucalyptus* species and “E. urograndis”, RLC_*egAle*_2 has similar expression characteristic in stalks from *Eucalyptus* species and “E. urograndis”. Families from the same lineage in sugarcane had distinct transcriptional pattern in leaves and buds [6].

High transcriptional levels in roots were described for several *Copia* LTR-RTEs families in different plant species, such as citrus [91] and *Quercus suber* [9].

This is the first work that LTR-RTEs were evaluated in roots submitted to PEG osmotic stress. *E. grandis* roots submitted to osmotic stress showed variable transcriptional activity. Three families with young insertion (<3 Mya) and with more genomic copies, RLC_*egIvana*_1, RLC_*egAle*_1 and RLC_*egAle*_2, had transcriptional activity modification, except RLC_*egMax*_1.

The transcriptional activity from RLC_*egAle*_1, RLC_*egAngela*_1, RLC_*egIvana*_1 e RLG_*egTekay*_1 families had a peak after 6 h of osmotic stress by PEG followed by a decrease in expression level in RLC_*egIvana*_*1* and RLG_*egTat*_1 families. This observation suggests that both families have their transcription triggered by similar stress conditions, a common feature among TEs [92]. Future functional studies validating the promoter specificity of these LTRs may shed a light on the stress activation of TEs in *Eucalyptus*.

## Conclusions

This study demonstrated that each *Copia* and *Gypsy* families in *Eucalyptus * have a different amplification pattern. Particularly in *E. grandis* and *E. urophylla*, that have diverged from a common ancestor ~ 20 Mya ago, we observed lower copy number in most LTR-RTEs at *E. urophylla* compared to *E. grandis*. These differences warrant further investigation to determine if recombination, nucleotide divergence or a specific burst of amplification can explain this pattern. Despite conservation to LTR-RTEs between species, IRAP and REMAP markers analyses based on transcriptionally active LTR-RTEs suggest different level of transpositional activity within *Eucalyptus* genus. This hypothesis is reinforced taking account that transcriptional activity is not the same among *Eucalyptus* species. Future studies can address if LTR-RTEs are specifically modulated by other stresses beside osmotic shock. Another important issue is to address if *Eucalyptus* LTR-RTEs families characterized here are in expansion in *Eucalyptus* genus, or even if they are conserved across other families rather than Myrtaceae, which may indicate horizontal transfer and/or purifying selection.

### Availability of supporting data

The data sets supporting the results of this article are available in the Dryad repository, doi:10.5061/dryad.h2t57.

## Additional files

Additional file 1: Tables S1-S6.
**Table S1.** Primers Sequences using different techniques from LTR-RTEs families. **Table S2.** PBS and PPT sequences from the LTR-RTE families characterized in *E. grandis* genome. **Table S3.** Diversity (Pi) between LTRs of retrotransposon families in Eucalyptus grandis genome. We used data from intact complete copies. **Table S4.** Total fragments and polymorphic bands in IRAP primers of five Eucalyptus species. **Table S5.** Total fragments and polymorphic bands in REMAP combinations of five *Eucalyptus* species. **Table S6.** Expressed sequence tags (EST) matched to complete LTR-RTEs elements. Additional file 2: Figures S1-S6.
**Figure S1.** RNA folding of RLC_*egAngela*_1 and RLC_*egAngela*_2 5’spacer region using RNAfold (http://rna.tbi.univie.ac.at/cgi-bin/RNAfold.cgi) with minimum free energy (MFE). The (a) RLC_*egAngela*_1 region 185-561; (b) RLC_*egAngela*_2 region 404-648. **Figure S2.** Distribution of full-length *Copia* and *Gypsy* elements across 11 chromosomes from *E. grandis*. Data was plotted using Circus. Tracks: (a) gene density (number per Mb, range 6 - 131) (Myburg *et al*. 2014); (b) Repetitive coverage (22 – 88% per Mb) (Myburg *et al*. 2014); (c) RLC_*egMax*_1 density (number per Mb, range 0 – 8); (d) RLC_*egBianca*_1 density (number per Mb, range 0 – 3); (e) RLC_*egAngela*_1 density (number per Mb, range 0 – 1); (f) RLC_*egAngela*_2 density (number per Mb, range 0 – 3); (g) RLC_*egIvana*_1 density (number per Mb, range 0 – 2); (h) RLC_*egAle*_1 density (number per Mb, range 0 – 2 p); (i) RLC_*egAle*_2 density (number per Mb, range 0 – 1); (j) RLG_*egTat*_1 density (number per Mb, range 0 – 2); (k) RLG_*egTekay*_1 density (number per Mb, range 0 – 2). **Figure S3.** IRAP analysis for a set of four *Eucalyptus* species using primers EgBiancaLTR-3 (a) and EgTatLTR-5 (b). L: 1 kb molecular weight marker: Gene Ruler DNA Ladder Mix (Fermentas). **Figure S4.** REMAP analysis for a set of three *Eucalyptus* species with primer Micro1 and EgAle2LTR-3 (a) or EgTatLTR-5 (b). L: 1kb molecular weight marker: Gene Ruler DNA Ladder Mix (Fermentas). **Figure S5.** UPGMA dendrogram of individuals from five *Eucalyptus* species using IRAP and REMAP data based on Jaccard distance.

## Abbreviations

TEs: Transposable elements
LTR-RTEs: LTR retrotransposons
ORFs: Open reading frames
Mb: Megabases
NCBI: National Center for Biotechnology Information
IRAP: Inter-Retrotransposon Amplified Polymorphism
REMAP: Retrotransposon-Microsatellite Ampliflied Polymorphism
TSDs: Target site duplications
MUSTER: MUlti-Sources ThreadER
MD: Molecular dynamics
GROMACS: Groningen Machine for Chemical Simulation
Rmsd: Root mean square deviation
UPGMA: Unweighted pair group method with arithmetic mean
qPCR: Quantitative real time PCR
INT1: RLG_*egTekay*_1 integrase
CD1: RLG_*egTekay*_1 chromodomain
RT1: RLG_*egTekay*_1 reverse transcriptase
INT2: RLC_*egAle*_1 integrase
RT2: reverse transcriptase of RLC_*egBianca*_1
