# Supplementary figures and images for: Transcriptionally active LTR retrotransposons in Eucalyptus genus are differentially expressed and insertionally polymorphic
Source: BMC Plant Biol. 2015 Aug 14;15:198. doi: 10.1186/s12870-015-0550-1 (PMC4535378; doi:10.1186/s12870-015-0550-1)

Figure S1

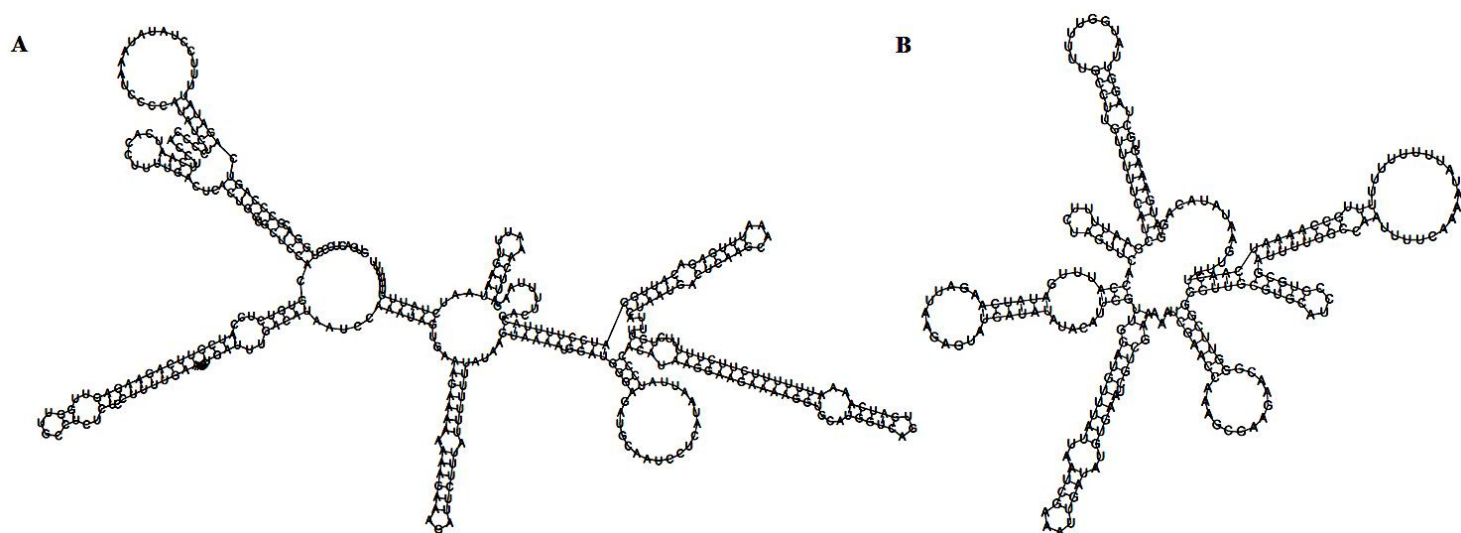

Figure S2

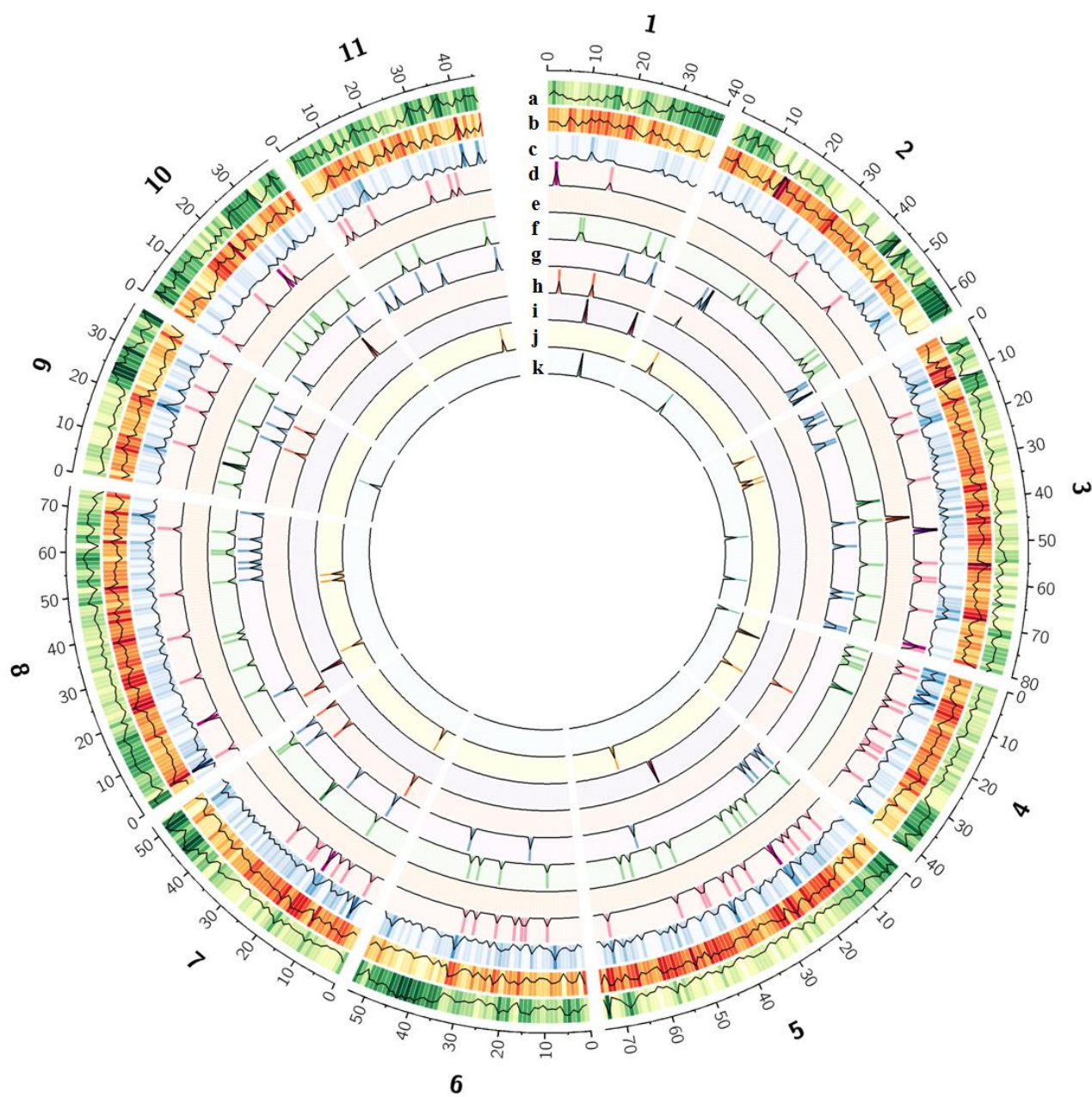

Figure S3

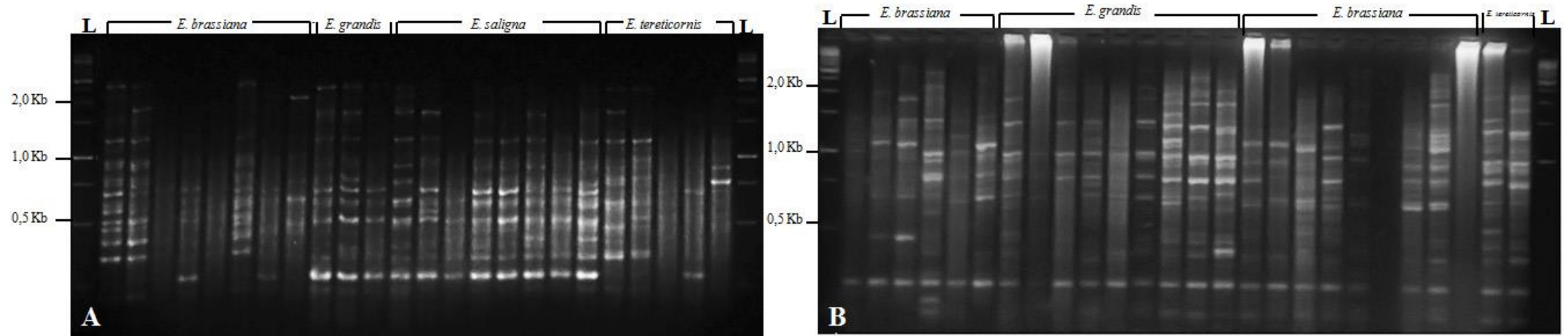

Figure S4

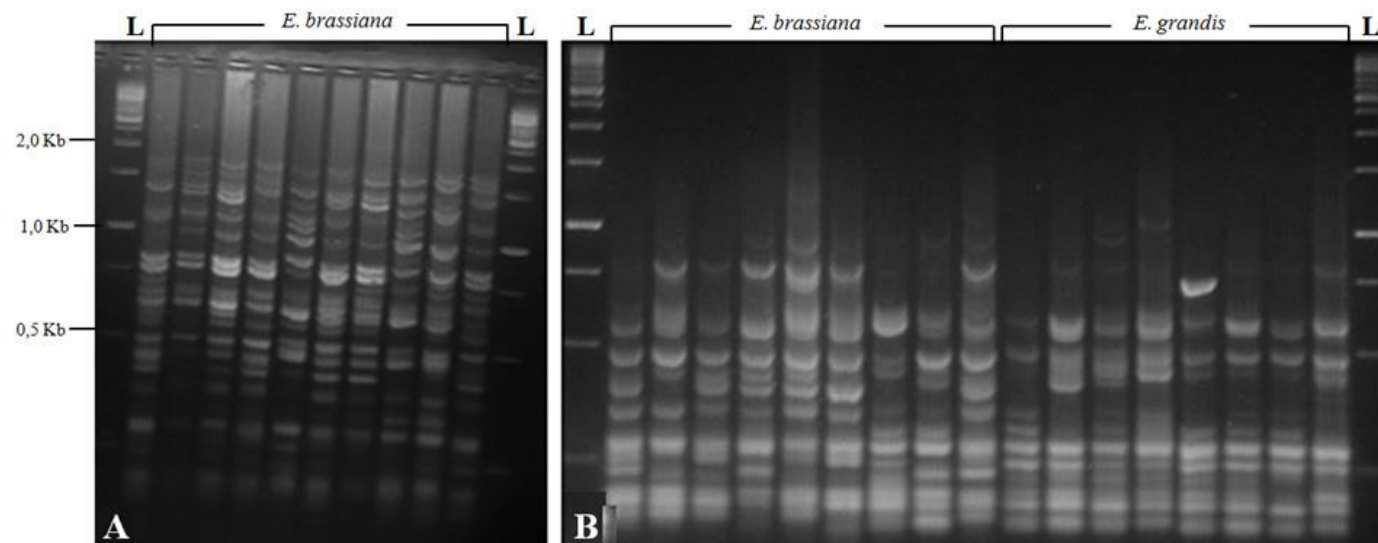

Figura S5

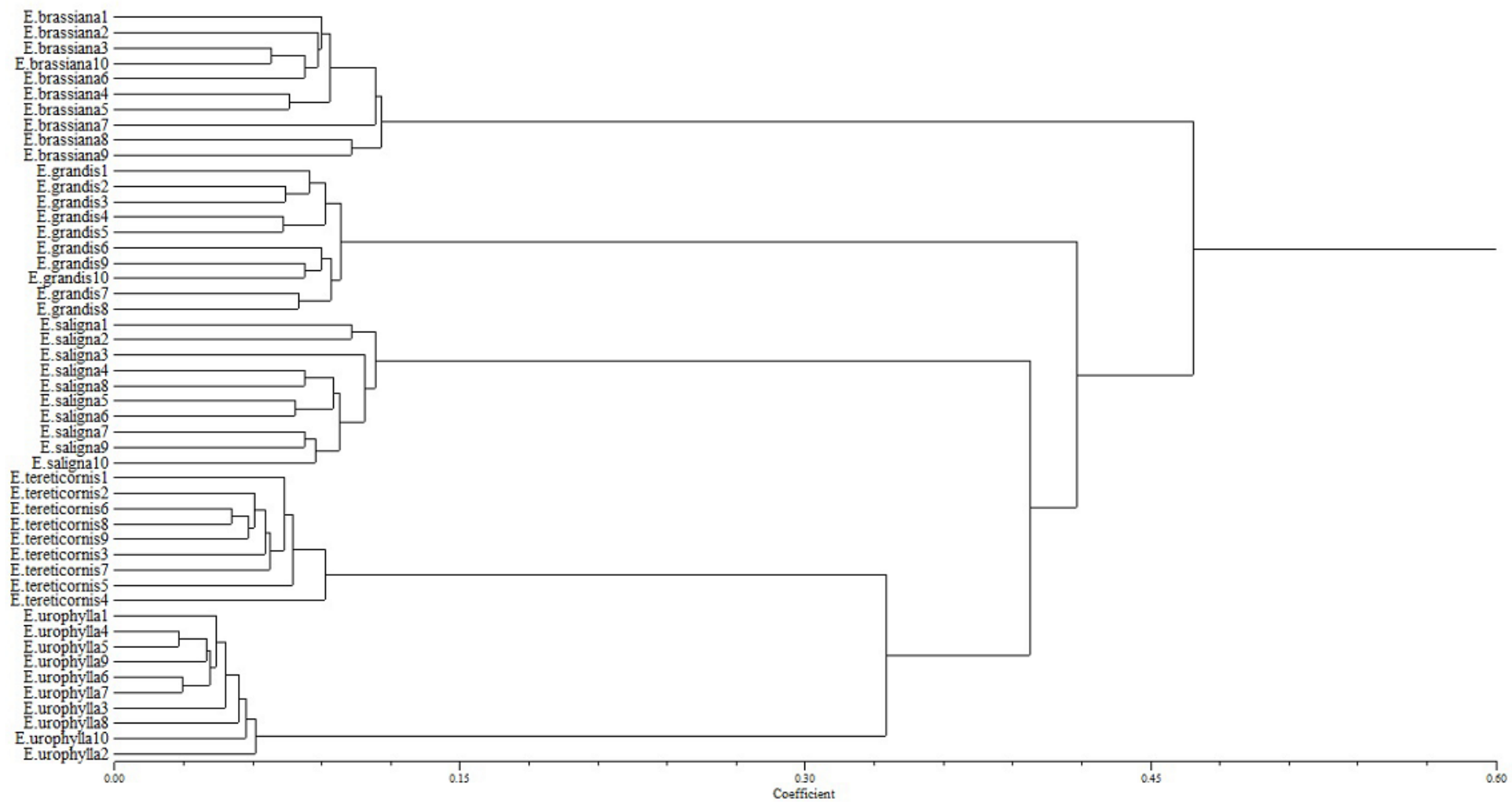

Supplement: Additional file 2: Figures S1-S6. — Figure S1. RNA folding of RLC_egAngela_1 and RLC_egAngela_2 5’spacer region using RNAfold (http://rna.tbi.univie.ac.at/cgi-bin/RNAfold.cgi) with minimum free energy (MFE). The (a) RLC_egAngela_1 region 185-561; (b) RLC_egAngela_2 region 404-648. Figure S2. Distribution of full-length Copia and Gypsy elements across 11 chromosomes from E. grandis. Data was plotted using Circus. Tracks: (a) gene density (number per Mb, range 6 - 131) (Myburg et al. 2014); (b) Repetitive coverage (22 – 88% per Mb) (Myburg et al. 2014); (c) RLC_egMax_1 density (number per Mb, range 0 – 8); (d) RLC_egBianca_1 density (number per Mb, range 0 – 3); (e) RLC_egAngela_1 density (number per Mb, range 0 – 1); (f) RLC_egAngela_2 density (number per Mb, range 0 – 3); (g) RLC_egIvana_1 density (number per Mb, range 0 – 2); (h) RLC_egAle_1 density (number per Mb, range 0 – 2 p); (i) RLC_egAle_2 density (number per Mb, range 0 – 1); (j) RLG_egTat_1 density (number per Mb, range 0 – 2); (k) RLG_egTekay_1 density (number per Mb, range 0 – 2). Figure S3. IRAP analysis for a set of four Eucalyptus species using primers EgBiancaLTR-3 (a) and EgTatLTR-5 (b). L: 1 kb molecular weight marker: Gene Ruler DNA Ladder Mix (Fermentas). Figure S4. REMAP analysis for a set of three Eucalyptus species with primer Micro1 and EgAle2LTR-3 (a) or EgTatLTR-5 (b). L: 1kb molecular weight marker: Gene Ruler DNA Ladder Mix (Fermentas). Figure S5. UPGMA dendrogram of individuals from five Eucalyptus species using IRAP and REMAP data based on Jaccard distance. [file 12870_2015_550_MOESM2_ESM.pdf]
